# Concurrent Radiation With Nivolumab and Chemotherapy for Nodal-Only Distant Metastatic Stage IV Esophageal Carcinoma

**DOI:** 10.7759/cureus.89807

**Published:** 2025-08-11

**Authors:** Mark E Bernard, Justin Rueckert, Jovan Z Pierre- Charles, Jordan Z Miller, Eddy Yang

**Affiliations:** 1 Radiation Medicine, University of Kentucky, Lexington, USA; 2 Pathology, University of Kentucky, Lexington, USA; 3 Cardiothoracic Surgery, University of Kentucky, Lexington, USA; 4 Radiation Medicine, University of Kentucky College of Medicine, Lexington, USA

**Keywords:** 18f-fdg pet/ct scan, definitive concurrent ctrt, esophageal carcinoma, immunotherapy, lymph node metastasis

## Abstract

The clinical response rates for patients with stage IV human epidermal growth factor receptor 2 (HER2) negative esophageal carcinoma treated with immunotherapy and chemotherapy remain low, around 10%. HER2 is a protein that is found on the surface of a cell, including cancer cells. This receptor can sometimes be targeted to help eradicate the cancer cell. Immunotherapy is a type of treatment that stimulates the immune system to eradicate other cancer cells in the body. Cancer cells often have a protein on their surface called programmed cell death ligand 1 (PD-L1). This protein binds to the programmed cell death 1 (PD-1) protein that is on white blood cells. The binding of these two proteins prevents the white blood cell from eradicating the cancer cell. Immunotherapy prevents the binding of these proteins and thus increases the chances of the white blood cell eliminating the cancer cell. The addition of concurrent radiation could improve this response rate by direct cell kill, increase the immunotherapy response via the abscopal effect, and eliminate micro-metastatic cancer. Obtaining a complete clinical response could be a prognostic marker for improved outcomes. A complete clinical response is where the imaging scans and/or direct visual examination of certain parts of the body show no evidence of cancer. We now present a unique case of concurrent radiation with nivolumab and chemotherapy for a distant nodal-only metastatic stage IV esophageal patient who obtained a complete clinical response within three months of his last radiation treatment.

## Introduction

Standard of care treatment for stage IV, locally advanced, or unresectable human epidermal growth factor receptor 2 (HER2) negative esophageal adenocarcinomas and esophageal squamous cell carcinomas consists of chemo-immunotherapy (CI) [1,2]. This paradigm is based upon the KEYNOTE-859 trial, which showed that pembrolizumab with chemotherapy resulted in a median overall survival (OS) and median progression-free survival (PFS) benefit when compared to placebo plus chemotherapy for HER2-negative locally advanced, unresectable, or metastatic esophageal adenocarcinomas [1]. The CheckMate 648 study also showed that both combinations of nivolumab plus chemotherapy and nivolumab plus ipilimumab are superior to chemotherapy alone for programmed cell death ligand 1 (PD-L1) 1% or greater, previously untreated, unresectable, advanced, recurrent, or metastatic esophageal squamous cell carcinoma. There are many ways patients can be categorized as unresectable or metastatic. One of them is due to patients having visceral and/or osseous metastasis. However, we have seen a unique case of a patient presenting with stage IV metastatic lymph-node only cancer with lymph node involvement typically beyond the surgical field. An example of this would be lymph node involvement in the retroperitoneal lymphatics below the renal veins. A thoracic surgeon may not want to operate on these patients. However, since we believe these patients have a unique biology, they may benefit from some form of local treatment to achieve disease-free survival or obtain a clinical complete response (cCR).

While KEYNOTE-859 showed a benefit of CI, the benefits were minimal [1]. CI resulted in a small improvement in median OS of 1.5 months (11.5 vs. 12.9 months), a slight improvement in median PFS of 1.3 months (5.6 vs. 6.9 months), and the complete response rate of only 9% [1]. This means there is room for improvement. The addition of radiation may improve outcomes due to the current published prospective trials. The Stereotactic Ablative Radiotherapy for the Comprehensive Treatment of Oligometastases (SABR-COMET) trial showed that the addition of SBRT to standard of care therapy for oligometastatic cancers led to a substantial median OS improvement of 22 months (28 vs. 50 months) and a good improvement in median PFS of 6.2 months (5.4 vs. 11.6 months) [3]. The ESO-Shanghai 13 trial showed that the addition of local therapy to systemic therapy, for patients with oligometastatic esophageal squamous cell carcinoma, resulted in a significant improvement in median PFS (6.4 vs. 15.3 months) [4]. Therefore, it is reasonable to assume that the addition of radiation therapy (RT) may result in a benefit for a subset of stage IV esophageal cancer patients.

Given the aforementioned data, we present a case report of a patient treated with concurrent CI and radiation.

## Case presentation

**Figure 1:**
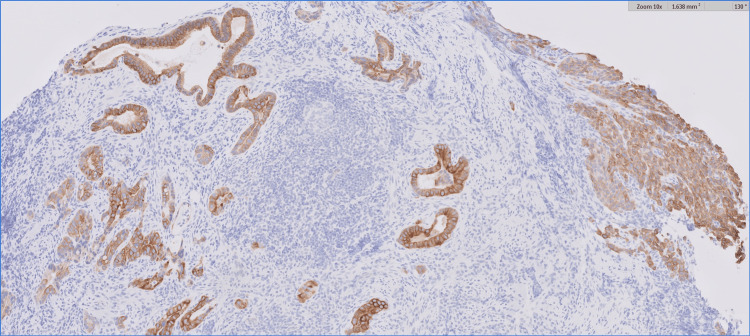
10× magnification showing strong CK7 positivity staining Focal gland formation is appreciated within the distal esophagus, which indicates an adenocarcinoma component, and the strong and diffuse CK7 positivity would also favor adenocarcinoma.

**Figure 2:**
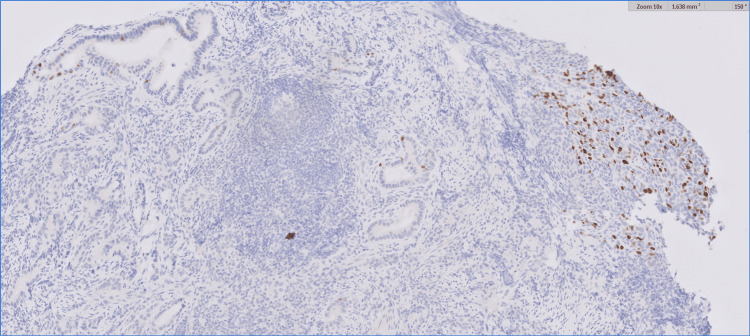
10× magnification showing patchy P40 positivity staining

**Figure 3:**
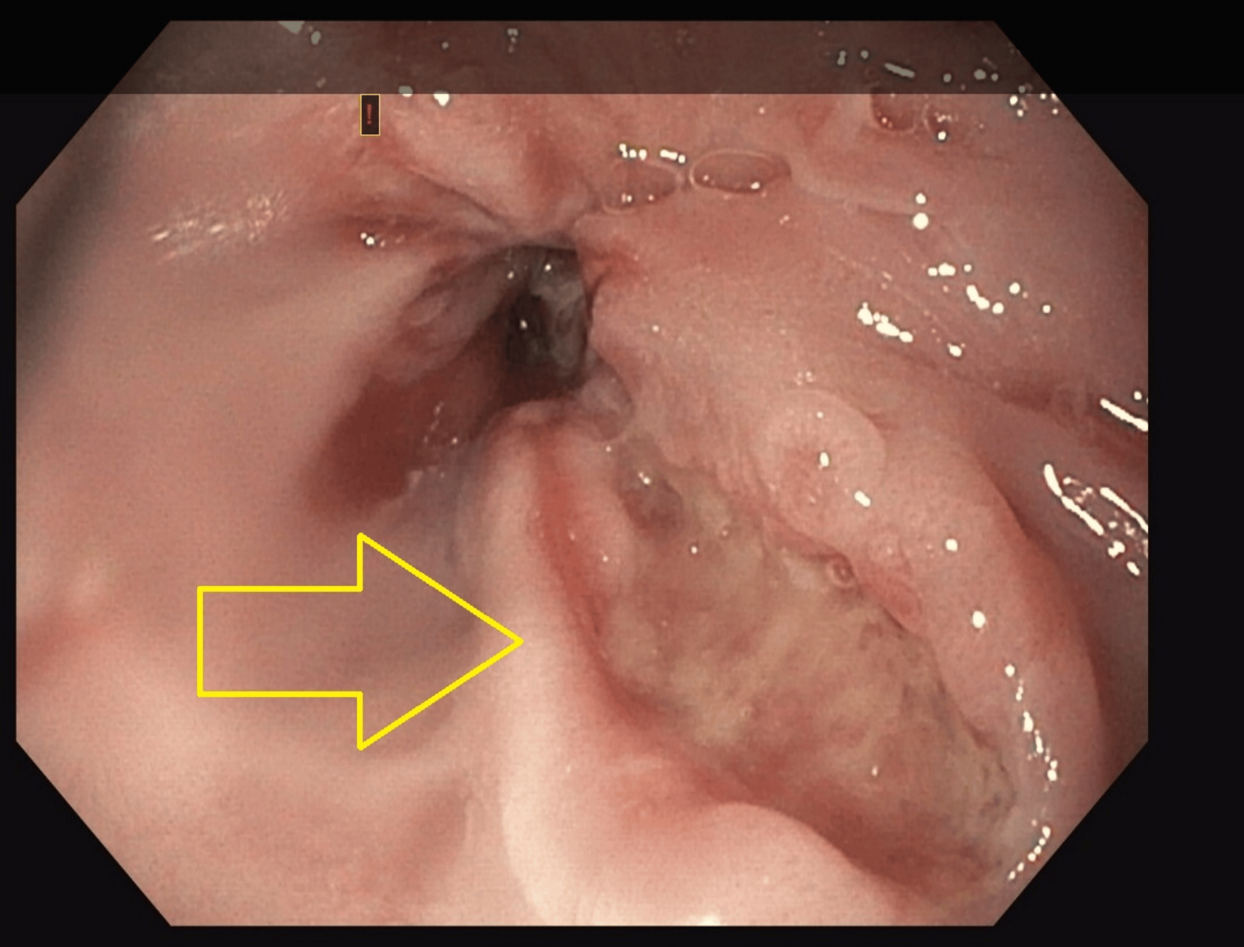
Esophagogastroduodenoscopy showing an ulcer in the distal esophagus The yellow arrows point to the ulcer in the distal esophagus.

Our patient presented as a 34-year-old male who had dysphagia for six months, mainly with solids, and was associated with frequent choking. This led to an esophagogastroduodenoscopy at an outside hospital, which showed a discrete ulcerated area occupying approximately 1/4 of the esophageal circumference located within 29-33 cm from the incisors. Subsequent biopsy of the distal esophagus showed poorly differentiated carcinoma with glandular features. His biopsy was later read at our institution and was reported as non-small cell carcinoma. PD-L1 had a combined positive score (CPS) of 5. HER2 was negative (1+), and microsatellite instability staining showed intact expression of all four proteins (MLH1, PMS2, MSH2, and MSH6). The biopsy showed strong CK7 staining, which indicates an adenocarcinoma; however, a squamous component could not be entirely excluded, given the patchy P40 staining. Pictures of the biopsy specimen are shown in Figures 1, 2. The pretreatment for an esophagogastroduodenoscopy is shown in Figure 3.

**Figure 4:**
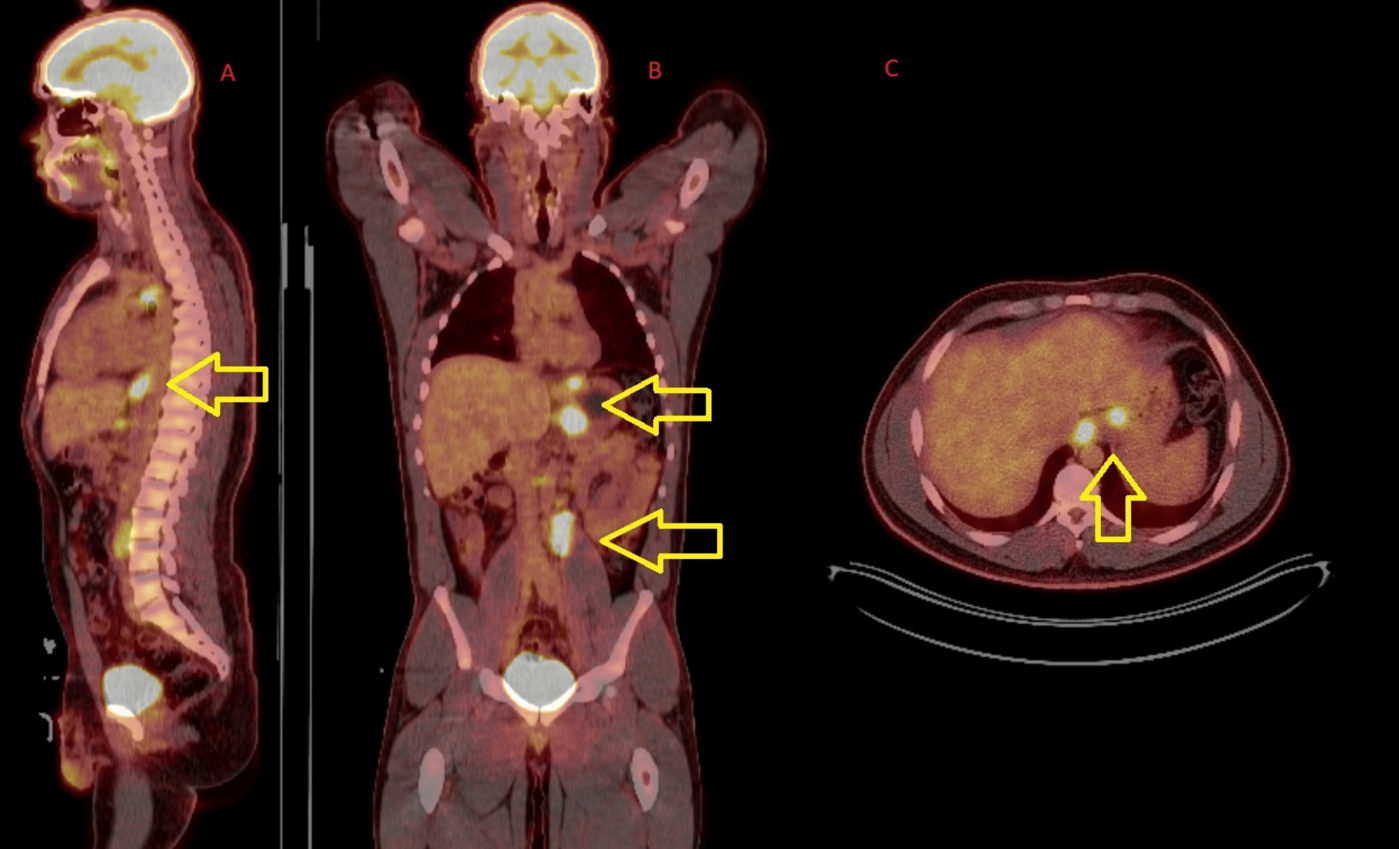
PET/CT scan showing a hypermetabolic distal esophageal adenocarcinoma primary with mediastinal, perigastric, and retroperitoneal lymph nodes The yellow arrows show the areas of hypermetabolic sites of cancer. (A) Sagittal view. (B) Coronal view. (C) Axial view.

Afterward, our patient had a PET/CT scan, which showed an intensely FDG-avid mass in the distal thoracic esophagus and multiple intensely FDG-avid mediastinal, gastric, and retroperitoneal lymph nodes consistent with distant nodal metastatic cancer and regional nodal spread. Pictures of this PET/CT scan are shown in Figure 4.

**Figure 5:**
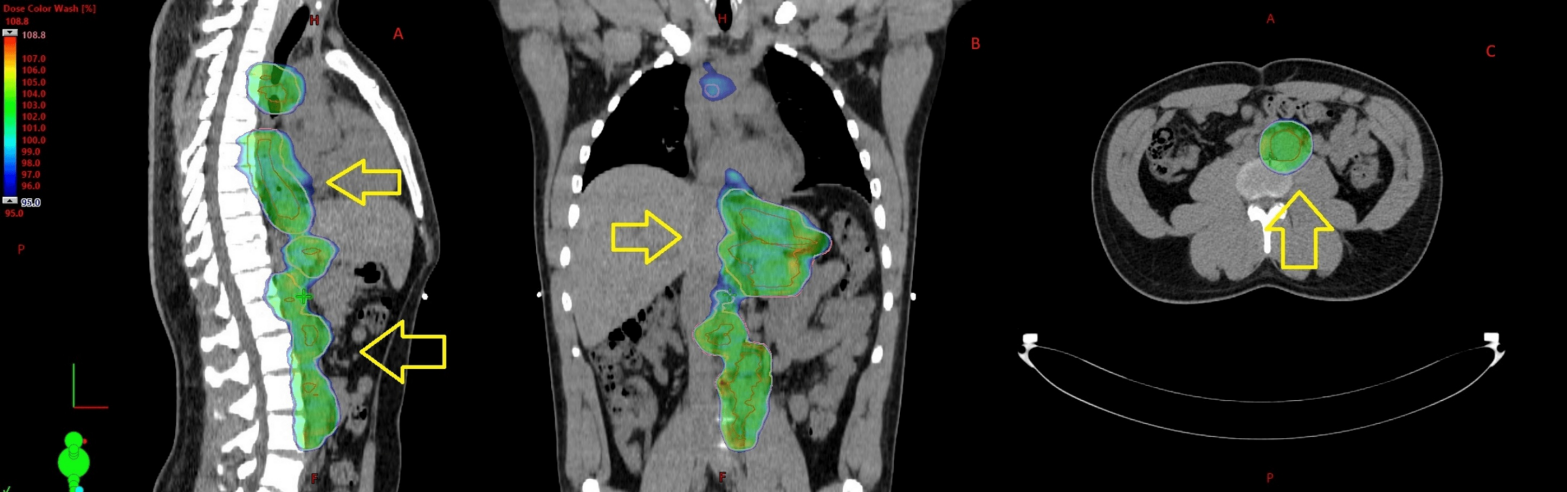
A dose color wash representation of his radiation plan targeting only the primary and the involved nodal sites The yellow arrows show that the 95% isodose line covers the planning target volume. The conformal nature of the 95% isodose line to the planning target volume is noted. (A) Sagittal view. (B) Coronal view. (C) Axial view.

He was evaluated by cardiothoracic surgery, medical oncology, and radiation oncology. Given his young age and distant metastatic cancer involvement being limited to the retroperitoneal lymph nodes, an esophagectomy may still be possible depending upon the response to systemic therapy and radiation. However, since his metastatic involvement made him stage IV yet being limited to nodal involvement only, the decision was made to treat with FOLFOX, nivolumab, and radiation therapy (RT). His FOLFOX and nivolumab followed standard dosing of every two-week infusion. His radiation treatment consisted of 46.8 Gy in 26 fractions to only the primary and metastatic nodal involvement. His planning target volume was 818.16 cm^3^. A picture of his radiation field and dose is shown in Figure 5.

During his treatment course, he developed Common Terminology Criteria for Adverse Events (CTCAE) grade 3 esophagitis requiring hospital admission and withholding of RT. However, he was eventually able to complete his course, albeit he was stopped early at 4680 cGy instead of 5040 cGy within 38 elapsed days. After completion of his RT, he went out to complete his systemic therapy, and at the time of creation of this manuscript, he continues on nivolumab.

**Figure 6:**
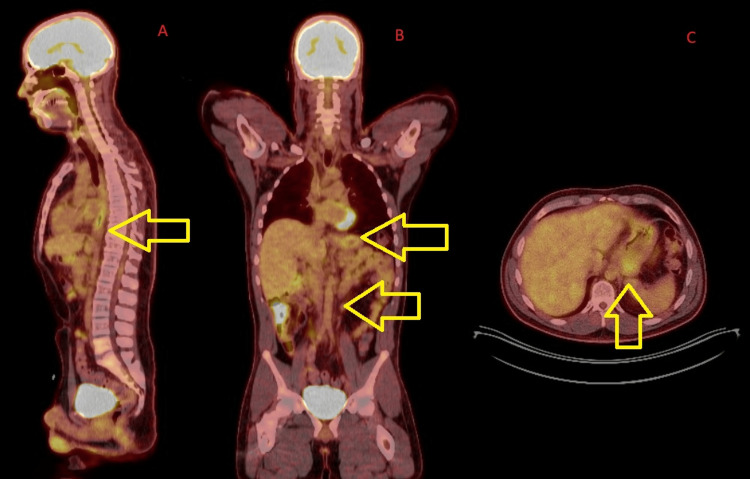
PET/CT showing complete metabolic response five months after completing concurrent chemo-immunotherapy with radiation The yellow arrows show the complete metabolic resolution of the previous sites of cancer. (A) Sagittal view. (B) Coronal view. (C) Axial view.

He also had a post-treatment EGD approximately seven weeks following his RT treatment, which showed no evidence of gross tumor in the distal esophagus with changes consistent with treatment effect. A post-treatment biopsy of the esophagus showed no definitive viable tumor within the biopsied material. Also, approximately five months after completion of his RT, he had a post-treatment PET/CT scan, which showed complete metabolic resolution of previously seen intense FDG uptake of the gastric and complete metabolic resolution of the hypermetabolic mediastinal and retroperitoneal lymph nodes. Therefore, he was able to achieve a complete clinical response. This is shown in Figure 6.

Sadly, approximately 12 months after his diagnosis, he was found to have a single brain metastasis, which was treated with craniotomy followed by Gamma Knife radiosurgery. However, he continued to show no extracranial evidence of recurrence.

## Discussion

This is the first case that we are aware of showing the feasibility of concurrent nivolumab, FOLFOX, and radiation in stage IV esophageal carcinoma with distant nodal spread. Our patient was able to achieve a cCR with this regimen and continued on single-agent nivolumab. This is a monumental outcome since complete responses are a rare phenomenon with systemic therapy alone. In the KEYNOTE-859 trial, which evaluated pembrolizumab plus chemotherapy, only 9% of patients in the intention-to-treat group had a cCR [1]. Even for patients with a PD-L1 CPS score of 10 or higher, the cCR was only 13%. In the CheckMate 648 trial, which evaluated nivolumab combination therapy in advanced esophageal squamous cell carcinoma, the cCR was 13% for the overall population and 16% for those with a tumor-cell PD-L1 expression of 1% or greater. Obtaining a cCR in esophageal cancer is a promising finding because these patients are reported to have a better prognosis [5]. In fact, patients with esophageal cancer who achieve a cCR with neoadjuvant chemoradiation and patients with rectal cancer who achieve a cCR after total neoadjuvant therapy can often omit surgery [5,6].

Achieving a cCR was most likely the result of direct local treatment with radiation, the cytotoxic efficacy of FOLFOX, and the immunomodulating effectiveness of nivolumab. However, the reason why no systemic progression occurred along with the cCR may be related to the synergistic response of immunotherapy and radiation. The combination of radiation and immunotherapy may have helped to prevent the progression of micro-metastatic cancer. RT can stimulate an immunotherapy response via the abscopal effect [7,8]. The abscopal effect starts with RT targeting the tumor, leading to the release of antigens and damage-associated molecular patterns (DAMPS). Antigen-presenting cells (APCs), such as dendritic cells (DCs), can pick up and process the tumor antigens to prime the CD8+ T-cells. The CD8+ T-cells are activated by DAMPs to upregulate co-stimulatory molecules necessary for CD8+ T-cell priming. These CD8+ T-cells are now primed against the TAs and are activated to eliminate other tumor cells with a similar or the same antigen at other sites [7,8]. Thus, RT can potentiate the immune system to attack other micro-metastatic and or macro-metastatic cancer sites. RT can also potentiate the immune response by activating the cytosolic DNA sensor cyclic GMP-AMP (cGAMP) synthase (cGAS) and the adaptor protein stimulator of interferon genes (STING) pathway [7,9]. The cGAS-STING pathway is a complex series of events that involves DCs detecting irradiated DNA and engulfing the irradiated tumor cells or internalizing irradiated tumor-derived exosomes, both of which eventually lead to the production of type-I IFNs [8]. The type-I IFNs bind to IFN-receptors on the DC, which stimulate DC activation and maturation. The activated DC primes the CD8+ T-cell with the TA by the DC using its MHC class I receptor to present the TA to the CD8+ T-cell receptor. The primed CD8+ T-cell is now able to recognize the TA-MHC I complex on the surface of other tumor cells and, once bound to other tumor cells, can induce apoptosis through either death receptor signaling, which involves binding of the CD8+ T-cell Fas ligand (FasL) with the tumor cell FAS receptor (FasR), or through the release of the granzyme B and perforin. In summary, RT can result in TA-primed CD8+ T-cells, TA-primed cross-presenting DCs, and the production of type I IFNs, all of which potentiate the ideal radiation-induced antitumor immune response.

As mentioned in the case report, he developed a single brain metastasis treated with resection followed by Gamma Knife radiosurgery; however, he continues to show no evidence of extracranial recurrence. His unique mode of recurrence is likely due to the blood-brain barrier limiting the white blood cell response to immunotherapy. As newer immunotherapy agents develop, which have better penetration through the blood-brain barrier, the benefit of radiation to concurrent CI can be potentiated.

## Conclusions

This case report potentially shows the feasibility of concurrent RT, nivolumab, and FOLFOX for stage IV esophageal carcinoma with distant nodal spread. We are currently awaiting the opening of our prospective trial to evaluate the feasibility of this regimen at the University of Kentucky, Lexington, KY.
